# Ischaemic stroke and Clostridium septicum sepsis and meningitis in a patient with occult colon carcinoma - a case report and review of the literature

**DOI:** 10.1186/s12883-016-0755-4

**Published:** 2016-11-24

**Authors:** Kosmas Macha, Antje Giede-Jeppe, Hannes Lücking, Roland Coras, Hagen B. Huttner, Jürgen Held

**Affiliations:** 1 Department of Neurology, Universitätsklinikum Erlangen, Friedrich-Alexander-Universität Erlangen-Nürnberg (FAU), Schwabachanlage 6, 91054 Erlangen, Germany; 2 Department of Neuroradiology, Universitätsklinikum Erlangen, Friedrich-Alexander-Universität Erlangen-Nürnberg (FAU), Schwabachanlage 6, 91054 Erlangen, Germany; 3 Department of Neuropathology, Universitätsklinikum Erlangen, Friedrich-Alexander-Universität Erlangen-Nürnberg (FAU), Schwabachanlage 6, 91054 Erlangen, Germany; 4 Mikrobiologisches Institut, Universitätsklinikum Erlangen, Friedrich-Alexander-Universität Erlangen-Nürnberg (FAU), Wasserturmstraße 3-5, 91054 Erlangen, Germany

**Keywords:** CNS infection, Pneumocephalus, Cerebritis, Meningoencephalitis, Sepsis, DIC, Ischemic stroke, Bacteremia

## Abstract

**Background:**

*Clostridium septicum* is a rare cause of meningitis and brain abscess in children and adults. Gas production by the pathogen can lead to pneumocephalus and the overall mortality rate of *Clostridium septicum* CNS infection is as high as 74%. The most common entry site of the pathogen is the gastrointestinal tract.

**Case presentation:**

We describe a 74-year-old man who presented with a left-sided cerebral infarction in the middle cerebral artery territory. In addition the patient showed signs of Systemic Inflammatory Response Syndrome and Disseminated Intravascular Coagulation. Examination of blood cultures and cerebrospinal fluid led to the diagnosis of sepsis and meningitis caused by *Clostridium septicum*. Despite appropriate antibiotic therapy the condition of the patient deteriorated rapidly and he died on day 2 after admission. Autopsy revealed a previously unknown adenocarcinoma of the *colon ascendens* as entry site of the pathogen.

**Conclusion:**

*Clostridium septicum* should be considered as potential pathogen in patients with sepsis and meningitis. Gram stain morphology in conjunction with severe sepsis can rapidly point into the direction of this pathogen. CNS infections manifest either as meningoencephalitis/cerebritis or as brain abscess. Entry site of the pathogen is almost uniquely the gastrointestinal tract. In adults more than 50% suffer from colorectal carcinoma, therefore survivors of *Clostridium septicum* infections should be examined for underlying occult colorectal malignancy.

## Background

The seven main pathogens causing community-aquired bacterial meningitis (*Streptococcus pneumoniae* (72%), *Neisseria meningitidis* (11%), *Listeria monocytogenes* (5%), *Haemophilus influenzae* (3%), *Streptococcus pyogenes* (2%), *Streptococcus agalactiae* (1%), *Staphylococcus aureus* (1%)) account for 96% of infections [1]. *Clostridium septicum* is a rare cause of central nervous system (CNS) infection. It manifests either as meningoencephalitis/cerebritis or as brain abscess and is associated with high morbidity and mortality [2, 3].

Here we describe a case of sepsis and meningitis caused by *Clostridium septicum* complicated with ischemic stroke and review the literature with a focus on pathogen, portal of entry, therapy and outcome.

## Case presentation

**Fig. 1:**
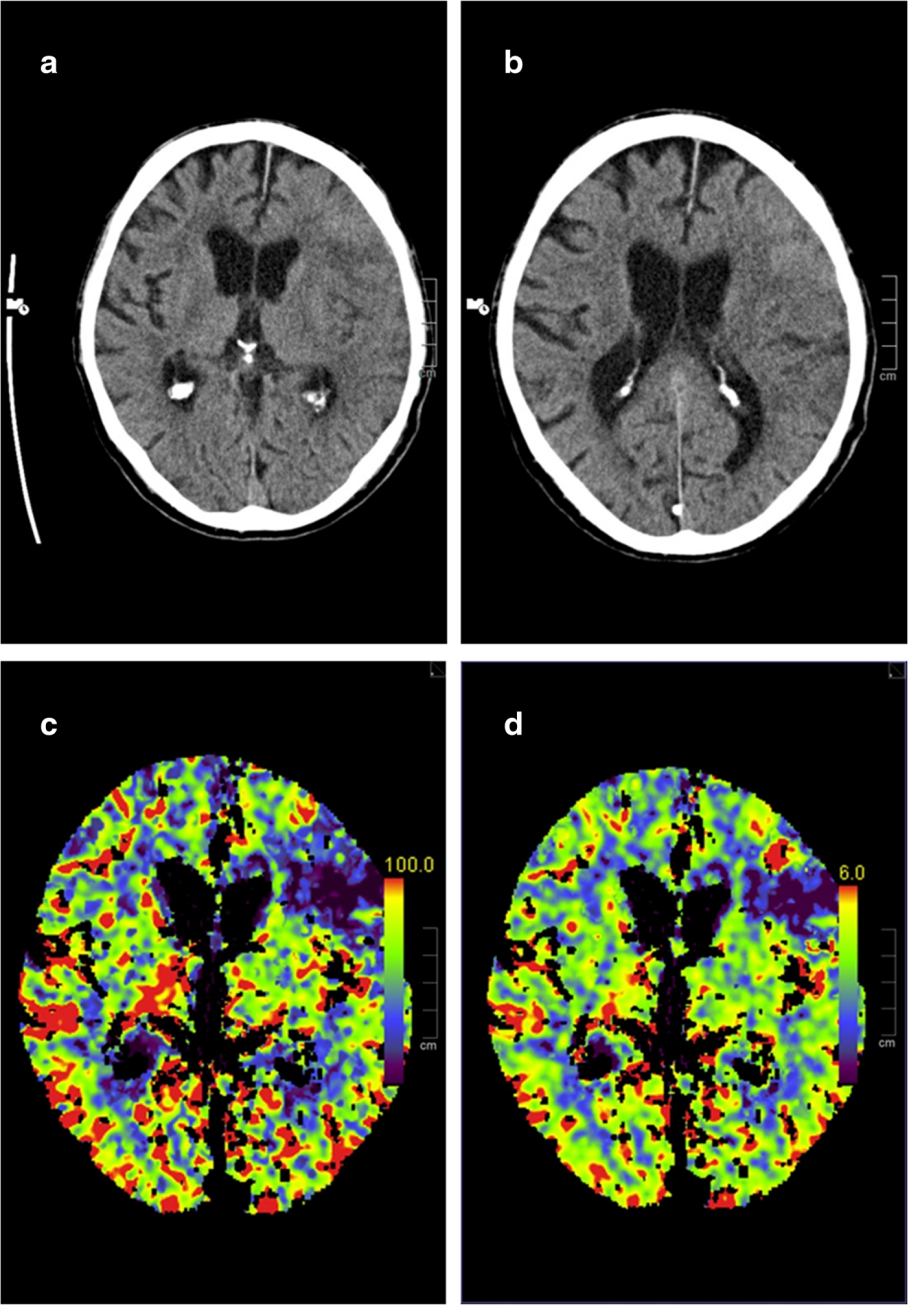
Non-contrast CT at admission showing left-sided MCA-infarction (**a**, **b**). CT-perfusion showing reduced perfusion of the left anterior MCA-territory in Cerebral Blood Flow (CBF)- (**c**) and Cerebral Blood Volume (CBV)-measurements (**d**)

**Fig. 2:**
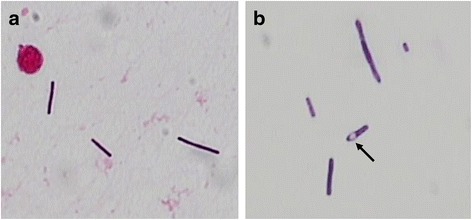
Gram stain morphology of *Clostridium septicum* from CSF (**a**) and blood culture (**b**) in 1000× magnification. Interestingly, spore formation (*arrow*) was only observed in Gram stains from blood cultures

A 74-year-old man was admitted to our emergency department in a somnolent state of consciousness with left gaze preference, aphasia, and right-sided hemiparesis (National Institutes of Health Stroke Scale, NIHSS-score: 17). The symptoms were first realized after awakening. Cranial computed tomography and angiography revealed a left-sided infarction in the middle cerebral artery (MCA) territory caused by a peripheral MCA occlusion (Fig. 1). In addition the patient showed signs of Systemic Inflammatory Response Syndrome (SIRS) and Disseminated Intravascular Coagulation (DIC) (body temperature 41.1 °C, heart rate 140/min, respiratory rate 17/min, leucocytes 4.17 × 10^3/ul, C-reactive protein (CRP) 54.3 mg/l, platelet count 111 × 10^3/ul). Intravenous fluid therapy was started immediately. After acquisition of blood cultures and cerebrospinal fluid (CSF), an empiric antibiotic therapy with ceftriaxone, ampicillin and aciclovir was initiated. Soon after, the patient required cardiopulmonary resuscitation in the setting of septic shock. After intubation and cardiopulmonary stabilization the patient was admitted to the neuro-intensive care unit. CSF examination showed 390 leucocytes/ml, elevated lactate (5.52 mmol/l) and protein levels (2783 mg/l) as well as normal glucose levels (66% of serum levels). Immediate Gram staining of CSF revealed partially elongated, Gram-positive rods without spore formation. Because of their size and appearance *Listeria* spp. could be ruled out and *Bacillus* spp. or *Clostridium* spp. were suspected (Fig. 2a). Therefore the antibiotic therapy was escalated with vancomycin. On the following day anaerobic blood cultures grew also Gram-positive rods with some of them showing endospore formation (Fig. 2b). Aerobic blood cultures remained sterile. Accordingly, sepsis and meningitis by *Clostridium* spp. were assumed. Culture results confirmed this tentative diagnosis and the bacteria were identified by mass spectroscopy as *Clostridium septicum*. Antibiotic susceptibility testing showed that the strain was sensitive to penicillin G, imipenem, clindamycin, vancomycin and metronidazole with minimal inhibitory concentration of 0.023 mg/l, 0.006 mg/l, 0.032 mg/l, 0.75 mg/l and 0.25 mg/l, respectively. Respiratory specimens and urine were unremarkable. Transthoracic echocardiography did not show endocarditis and there was no sign of an infection focus in ear, nose, throat or the sinuses. The further course was characterized by a persistent severe septic disease pattern with high demand of catecholamines, strong increase of inflammation parameters (leucocytes max. 42.86 × 10^3^/ul, procalcitonin (PCT) max. 177.32 ng/ml, CRP max. 398.8 mg/l). The antibiotic therapy was switched to high dose penicillin G and clindamycin. The inflammatory parameters started to decrease approximately 48 h after initiation of empiric antibiotic therapy. However, because the patient showed a persistent loss of brain stem reflexes after initial cardiopulmonary resuscitation, it was decided to change the therapy to a palliative regimen. The patient died two days post admission. Due to the fact that no obvious risk factors for *Clostridium septicum* bacteremia were present (e.g. infected wounds, myonecrosis, diabetes mellitus, severe atherosclerotic cardiovascular disease, neutropenia, liver cirrhosis), a previously unknown gastrointestinal focus of infection was most likely [4]. Accordingly, autopsy revealed an adenocarcinoma of the colon ascendens with 2.8 cm in diameter as entry site of the pathogen.

## Discussion

The leading neurological symptoms of our patient were those of a left sided stroke in the MCA-territory which was confirmed by cranial computed tomography and angiography.

At the same time the patient suffered from bacterial sepsis and meningitis. The question in dispute is what came first? One order of events is that *Clostridium septicum* crossed the gastrointestinal barrier and entered the blood stream with the colon carcinoma as entry site. From the blood it spread to the CNS causing meningitis. In parallel, the sepsis lead to a DIC which on its part resulted in hypercoagulation and cerebral infarction. The stroke would have been a consequence of invasive clostridial infection.

A second possibility is that the stroke was the initial event leading to a compromised immune response and/or by stress-mediated paralytic ileus to an impaired intestinal barrier [5]. This enabled *Clostridium septicum* to enter the blood stream and supported by the stroke-induced damage of the blood-brain barrier (*locus minoris resistentiae*) to cause CNS infection. The invasive clostridial infection would have been a consequence of the stroke.

Two facts argue against the second hypothesis. First, the stroke was a wake-up stroke and sepsis and DIC were already apparent at admission. Therefore, the stroke induced impairment of the intestinal barrier must have developed within a couple of hours which is rather unlikely. Second, no bacteria were detected microscopically in the stroke area during brain autopsy and the published cases of *Clostridium septicum* CNS infections showed that the pathogen does not need an impaired blood-brain barrier to cause meningitis.

In our opinion the first hypothesis is more likely although we cannot rule out the second sequence of events with certainty.

The first hint on the nature of the pathogen was its CSF Gram stain morphology. All the major pathogens can be distinguished by their different appearance in the Gram staining. Only *Listeria monocytogenes* is Gram-positive and rod-shaped. However, size and shape of the Gram-positive rods in our patient pointed towards *Bacillus* or *Clostridium* spp. as pathogens. To cover possible *Bacillus*-infection the addition of vancomycin to the empiric antibiotic regimen with ampicillin and ceftriaxon was reasonable.

Clostridia are obligately anaerobic, gram-positive rods. Because of their ability to produce environmentally resistant endospores they are widespread in nature and are commonly found in soil and the intestinal tracts of humans and animals. Clostridia produce more kinds of protein toxins than any other bacterial genus causing diseases like tetanus, botulism, enterocolitis and anaerobic myonecrosis (gas gangrene). One important species is *Clostridium septicum*. Due to production of its 4 major exotoxins (α-, β-, γ- and δ-toxin) it is able to cause myonecrosis. In line with *Clostridium perfringens* and *Clostridium tertium* it is the third most common *Clostridium* spp. causing sepsis [4]. Patients with bacteremia by this organism are usually severely ill and mortality rates rise up to 70% [3]. More than 50% suffer from some kind of gastrointestinal pathology, such as diverticular disease, or an underlying malignancy, such as colorectal carcinoma. Further risk factors for clostridial bacteremia include neutropenia with or without enterocolitis, diabetes mellitus or gas gangrene [4]. Whether *Clostridium septicum* colonizes the human gastrointestinal tract is still under debate. However, a breach in the mucosal barrier for example by enterocolitis or colorectal cancers is required for invasive infection [3].

A rare complication of *Clostridium septicum* bacteremia is a hematogenous spread to the CNS. CNS infections manifest either as meningoencephalitis/cerebritis or as brain abscess [2]. Because of the ability of *Clostridium septicum* to produce gas a pneumocephalus may result that can be localized or diffuse [6–8]. Brain autopsy in our patient confirmed a haemorrhagic infarction in the left MCA territory. There were signs of diffuse inflammation at supra- and infratentorial areas in terms of a meningoencephalitis. An infectious agent and/or gas production could not be verified at microscopic level.

Table 1 summarizes the 19 published cases of *Clostridium septicum* CNS infections. 79% of the patients were either younger than 3 or older than 65 years of age. Nearly all affected children had hemolytic uremic syndrome probably after gastrointestinal infection with Enterohemorrhagic *Escherichia coli* and more than half (63%) of the patients older than 65 years suffered from colorectal carcinoma.

**Table 1:** Summary of published *Clostridium septicum* CNS infections

First Author	Age [years]	Sex	CNS manifestation	Underlying disease	Outcome	Day of death after admission
Bhogal P [3]	80-89	female	Meningoencephalitis with pneumocephalus	Gastroenteritis	Died	1
Broughton RA [10]	2	female	Meningoencephalitis	HUS	Died	1
Cheng YT [2]	40	female	Brain abscess	Multiple wounds, breast cancer	Survived	-
Chiang V [11]	2	male	Brain abscess	HUS	Survived	-
Dirks C [12]	72	female	Meningoencephalitis with pneumocephalus	Myonecrosis	Died	1
Gorse GJ [13]	76	male	Meningoencephalitis with pneumocephalus	Colorectal carcinoma	Died	1
Gorse GJ [13]	33	male	Meningoencephalitis with pneumocephalus	Aplastic anemia with splenectomy	Died	2
Habscheid W [14]	55	male	Meningoencephalitis with pneumocephalus	Neutropenic enterocolitis	Died	2
Macha K*	74	male	Meningoencephalitis	Colorectal carcinoma	Died	3
Katyal A [6]	66	male	Meningoencephalitis with pneumocephalus	Unknown	Died	1
Kolbeinsson MW [15]	71	male	Brain abscess	MDS	Survived	-
Mrangou AG [16]	85	male	Brain abscess	Colorectal carcinoma	Died	16
Martin SE [7]	2	male	Meningoencephalitis with pneumocephalus	HUS	Died	1
Ragland RL [17]	67	male	Meningoencephalitis with pneumocephalus	Colorectal carcinoma	Died	1
Randall JM [8]	4	male	Meningoencephalitis with pneumocephalus	HUS	Died	3
Riccio JA [18]	1	male	Meningoencephalitis with pneumocephalus	HUS	Died	1
Roeltgen D [19]	73	female	Meningoencephalitis with pneumocephalus	Colorectal carcinoma	Died	1
Sadarangani SP [20]	1 month	male	Brain abscess	Necrotizing enterocolitis	Survived	-
Williams EJ [21]	2	male	Brain abscess	HUS	Survived	-

*This patient is described in the present case report

According to our literature review the overall mortality in patients with *Clostridium septicum* CNS infection is 74% (5 survivors, 14 deceased) and most patients died within the first 48 h (79%). Interestingly, the mortality of brain abscess with the possibility of a surgical intervention is significantly lower than that of meningoencephalitis with antibiotic therapy alone (17% versus 100%, *p* = 0.001).

Therefore, therapeutic management of *Clostridium septicum* CNS infection consists of antibiotic therapy and, if possible, surgery/aspiration of the brain abscess. Antibiotics with low MICs are penicillin G, cefazolin, clindamycin and rifampin. A combination therapy of penicillin plus clindamycin or rifampin might be beneficial [9]. Prognosis of meningoencephalitis with antibiotic therapy alone is poor and it seems essential for survival that a localized process (e.g. an abscess) exists that is accessible to surgical interventions.

## Conclusions

*Clostridium septicum* should be considered as potential pathogen in patients with sepsis and meningitis. Gram stain morphology in conjunction with severe sepsis can rapidly point into the direction of this pathogen. Detailed microbiological analysis is required for identification and appropriate antibiotic therapy. If a localized process in the CNS is present a surgical intervention seems to be essential for survival. Adult patients with *Clostridium septicum* infection should be examined for underlying occult gastrointestinal disease or cancer.

## Abbreviations

CNS: Central nervous system
CRP: C-reactive protein
CSF: Cerebrospinal fluid
DIC: Disseminated intravascular coagulation
EHEC: Enterohemorrhagic *Escherichia coli*
HUS: Hemolytic uremic syndrome
MCA: Middle cerebral artery
MDS: Myelodysplastic syndrome
NIHSS: National Institutes of Health Stroke Scale
PCT: Procalcitonin
SIRS: Systemic Inflammatory Response Syndrome
